# A Novel Pediatric Clinical Skills Curriculum to Prepare Medical Students for Pediatrics Clerkship

**DOI:** 10.1007/s40670-024-02191-w

**Published:** 2024-11-13

**Authors:** Lindsay C. Podraza, Lauren S. Starnes, Joseph R. Starnes, Anuj Patel, Rachel K. P. Apple

**Affiliations:** 1 Monroe Carell Jr. Children’s Hospital at Vanderbilt, Nashville, TN USA; 2 Vanderbilt University Medical Center, Nashville, TN USA

**Keywords:** Near-peer learning, Flipped-classroom model, Infant manikin, Pediatric physical examination skills, Pediatric clinical skills, Clerkship preparedness, Preclinical pediatrics curriculum

## Abstract

**Introduction:**

Medical students feel poorly prepared to examine pediatric patients during clerkship. Our institution’s introduction to clinical skills course lacked practice with pediatrics physical examination skills. We developed a novel clinical skills curriculum to increase students’ confidence in examining pediatric patients.

**Methods:**

Ericsson’s deliberate practice conceptual framework guided curriculum design. We utilized a flipped-classroom model to teach the newborn examination. Students watched a video, then practiced with manikins and patients. For the child examination, students attended a lecture and practiced with hospitalized children and facilitators. Students then participated in a Home, Education, Eating/Exercise, Activities/Employment, Drugs, Suicidality, Sexuality, Safety (HEEADSSS) didactic and role play activity. Before and after participation, students completed REDCap surveys ranking confidence in performing pediatric examinations and identifying normal examination findings on a Likert scale (1 = “Not at all confident,” 4 = “Extremely confident”). We analyzed data using Wilcoxon rank sum tests.

**Results:**

A total of 97 students participated in the curriculum. Respectively, 56 (58%) and 32 (30%) students completed pre- and post-participation surveys. Post-participation, students reported increased confidence in identifying normal infant (median [interquartile range]; (2 [2,2] vs 4 [3,4]; *p* < 0.001) and child (2 [2,2] vs 3 [3,4]; *p* < 0.001) examination findings as well as HEEADSSS assessment components (2 [1.5,2] vs 4 [3,4]; *p* < 0.001), and had significantly higher scores on confidence performing infant (2 [2,2.5] vs 4 [3,4]; p < 0.001), child (2 [2,2] vs 3 [3,4]; *p* < 0.001), and HEEADSSS assessment (2 [2,3] vs 4 [3,4]; *p* < 0.001).

**Discussion:**

This multi-modal curriculum emphasizing pediatric examination skills improved students’ confidence in pediatric-specific knowledge and skills prior to clerkship.

**Supplementary Information:**

The online version contains supplementary material available at 10.1007/s40670-024-02191-w.

## Introduction

In transitioning to clerkship, many medical students feel poorly prepared to assess a patient based on physical examination [1]. In addition, a recent study reported that one-third of surveyed medical students felt that their preclinical preparation for the pediatrics clerkship was “fair” or “poor” [2]. While there were significant deficits in levels of preparedness across all categories (overall clerkship preparedness, communication, physical examination, and medical knowledge), 40% of students felt underprepared on pediatric physical examination skills when compared to the other core clerkships [2]. Notably, students pointed out lack of practice with the newborn examination as a perceived gap in their preclinical education and reported the desire to interact with real children prior to clerkship [2]. When clinical skills course directors and pediatric clerkship directors were surveyed, the majority expected students to have experience with these skills prior to beginning their pediatrics clerkship [1]. While some disagreements about where and when these skills ought to be taught exist, it should be noted that time spent on clerkships in general has decreased over the last 10 years [1]. Time on the pediatrics clerkship is limited even further, with the national average being 6.6 weeks [1]. Thus, integrating pediatric-specific clinical skills teaching into the pre-clerkship (traditionally adult-centric) clinical skills curriculum is essential for ensuring adequate pediatric-specific learning opportunities for medical students.

A major barrier to teaching pediatric physical examination skills is finding children and infants to participate, as standardized patients are usually limited to adults. Some studies have investigated the use of infant manikins to combat this [3, 4]. However, students also desire interaction with real infants and children, as this is often a point of hesitation upon starting their pediatrics clerkship [2].

Another obstacle for students is the Home, Education/Employment, Eating, Activities, Drugs, Sexuality, Suicidal ideation, Safety (HEEADSSS) assessment for adolescent patients, which is a psychosocial screening tool that can allow for discovery of major causes of morbidity and mortality among this population through identification of risky behaviors [5, 6]. Takeuchi et al. identified several hindrances for students faced with interviewing an adolescent patient, including their own preconceptions about this population, lack of experience with this age group, and difficulties managing professional distance when they consider themselves close in age or socially similar [6]. Unfortunately, time spent teaching adolescent medicine topics is limited in both graduate and post-graduate training, and literature on the approach to teaching adolescent medicine to pre-clerkship trainees remains scarce. As a result, future physicians may feel uncomfortable addressing this population’s needs in practice [7].

Preclinical exposure to pediatrics remains a sparsely populated area within medical education literature, and there are only limited reports of curricula designed to remedy the lack of pediatrics content in this domain. However, the limited studies that do exist have demonstrated significant benefits, including improved pediatrics-specific knowledge, skills, and communication technique, as well as increased preparedness for pediatrics clerkship and confidence working with the pediatrics population [8–11]. We aimed to perform a problem assessment and create a theory-based educational intervention to improve medical student confidence in performing pediatric physical examinations and recognizing normal findings, as well as understanding and performing the HEEADSSS assessment.

A systematic review on physical examination teaching by Mookherjee et al. concluded that Ericsson’s deliberate practice conceptual framework is beneficial in teaching physical examination skills [12]. This framework emphasizes several key principles in working towards gaining expertise, including defining a specific goal, receiving and responding to high-quality feedback from expert guidance, and providing repeated opportunities to practice the skill to improve over time [13]. Using these principles, we implemented several in-person sessions focused on skills acquisition using near-peers in a small group model to increase opportunities for practice and feedback. Furthermore, the literature suggests that small-group learning allows for peer-to-peer observation and thus greater acquisition of skills [14]. In a busy clinical setting that makes the ideal small student-to-teacher ratio difficult to achieve, near-peer teachers can be utilized as an effective way to optimize this ratio and increase learning opportunities for students [15]. An added benefit of the near-peer model is increased knowledge and confidence in skills for both medical student and teacher [15]. To maximize time spent actively honing skills with the near-peer small group model, we employed the flipped-classroom approach, which has been shown to be an effective method for teaching the physical examination [16]. Our outcomes primarily focused on Kirkpatrick’s second level of assessment — how learning impacts student confidence [17].

## Methods

Using Kern’s six-step approach, we first performed a general problem assessment within our clinical skills curriculum [18]. At our institution, first-year medical students complete this 14-month preclinical longitudinal curriculum in preparation for clerkship rotations the following year. The curriculum starts in July of their first year of medical school. Content within the clinical skills curriculum mirrors content in the basic science courses that students are simultaneously enrolled in. For example, when students are learning about the physiology and pathophysiology of the cardiovascular system in their basic science course, the clinical skills course teaches cardiovascular examination skills. We identified a significant problem in that our clinical skills course lacks a pediatric-specific curriculum and includes very minimal hands-on practice with pediatric patients. For example, the current course fails to address the newborn examination, as this topic historically has been deferred to the short time students spend in the newborn nursery during their clerkship. Additionally, it lacks dedicated teaching on the HEEADSSS assessment. We implemented our pediatrics clinical skills curriculum in the spring semester over the course of six weeks during which time students were engaged in their reproductive and endocrine basic science blocks. This is because we wanted them to have basic history and examination skills before learning the pediatric version of these elements, and because the basic science course covers typical hormonal development and puberty during this block, which is relevant to pediatrics. To pass the clinical skills course, students must average 70% or higher on all assessments and pass a final OSCE-style practical examination.

**Fig. 1:**
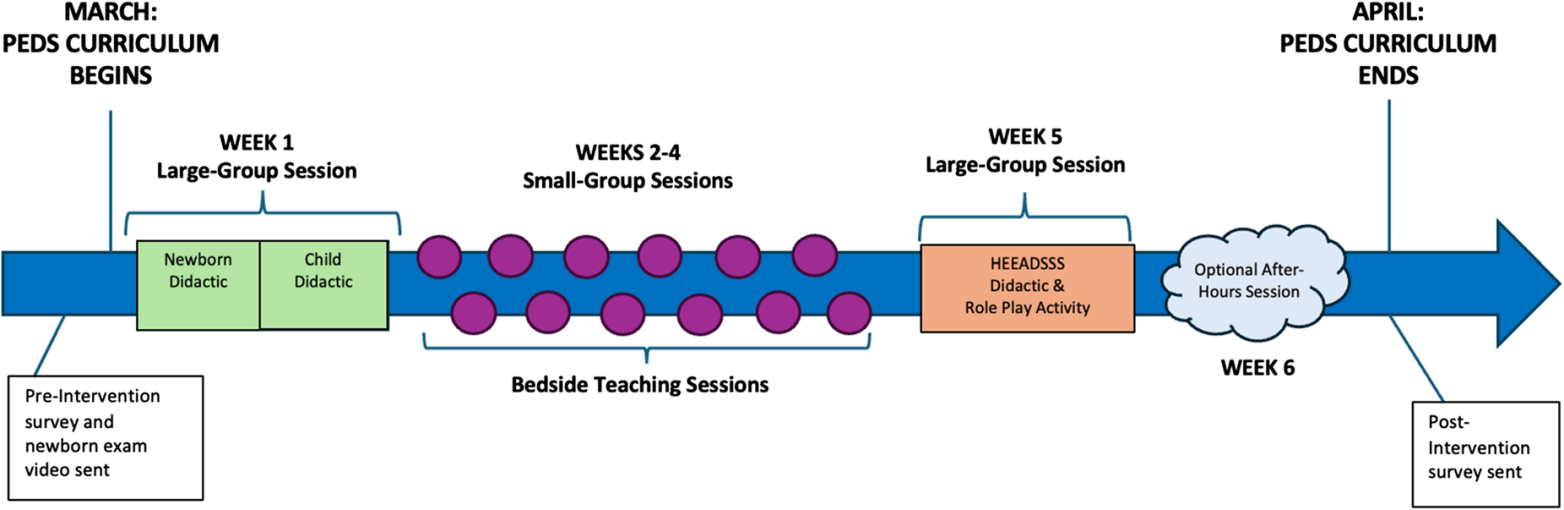
Pediatrics curriculum schematic with timeline of scheduled activities as described in the text. Of note, this schematic represents the pediatrics clinical skills curriculum that takes place during months of March and April, within the 14-month longitudinal clinical skills curriculum that starts in July of the first academic year

To encompass the broad range of pediatric patient ages, we designed interventions aimed at the following pediatric patient groups: newborn/infant, child, and adolescent/teenager. Taken together, our curriculum required students to attend three one-hour didactic sessions and one one-hour bedside session (Fig. 1). Of note, all sessions took place within the children’s hospital to further immerse students in the care of pediatric patients. Our children’s hospital is a tertiary care center with 343 beds connected to a larger medical center in a metropolitan area. Our objectives consisted of the following: after participation in this curriculum, learners will be able to report increased confidence in systematically examining a healthy newborn/infant and child, recognizing normal newborn/infant and child examination findings, identifying components included in a HEEADSSS assessment, and performing a HEAADSSS assessment.

### Newborn/Infant

Prior to the first session, students were sent a 15-minute online video of a newborn nursery nurse practitioner performing the newborn examination and were instructed to watch it at home (Appendix A).

On week 1, the course officially began with a two-hour large-group session (green boxes, Fig. 1). Given the large number of students, the class was split in half and separated into different rooms. Each group participated in simultaneous sessions on newborn/infant and child examination teaching and swapped rooms at the end of the first hour.

For the first 15 minutes, a resident gave a brief overview of the newborn examination via a PowerPoint presentation (Appendix B) [19–24]. Students were then broken up into groups of four to six with a facilitator. Facilitators consisted of near-peers (residents) and faculty. Facilitators were expected to be proficient with the newborn examination prior to leading a session. Each group was provided with either a JIZH realistic newborn baby doll or infant manikin simulator in addition to a newborn examination checklist (Appendix C) to promote standardization of the experience across all groups. Facilitators were instructed to demonstrate a head-to-toe examination, and then encourage students to take turns practicing on the doll/manikin while receiving real-time feedback from the facilitator. Students not performing the examination were instructed to remain in their groups to observe their peers. Students were offered the opportunity to interact with both the dolls and manikins during the session.

### Child

Students participated in a traditional didactic presentation on the head-to-toe examination of a child that already existed within the curriculum. Two physician parents gave this PowerPoint-format didactic and demonstrated physical examination maneuvers on their children.

### Bedside Teaching

During weeks 2–4, students each participated in a one-hour small-group bedside teaching session with near-peer facilitators (residents and fourth-year medical students) (purple circles, Fig. 1). Groups were provided with the same checklist that was utilized during the first newborn/infant session (Appendix C), in addition to a child examination checklist (Appendix D) to promote standardization of the experience. Facilitators were expected to be proficient in the examination prior to the session and were responsible for identifying pediatric patients in the hospital who would be good candidates for student practice. Students took turns practicing maneuvers on infants and children while receiving real-time feedback during the encounter. To ensure that all students had the chance to participate, bedside sessions were offered 12 times throughout the course (six dates with two one-hour sessions each). Students were instructed to attend the session that fit into their schedule. Attendance was tracked using an online sign-up sheet that facilitators checked at each session.

### Adolescent/Teenager

During week 5, students attended an additional one-hour large-group session on a practical approach to the HEEADSSS assessment (orange box, Fig. 1). The first half of the session consisted of a resident-led PowerPoint presentation on the HEEADSSS assessment components (Appendix E) and suggested approaches to asking sensitive questions [25–28]. The second half of the session included a role-play activity using patient scripts (Appendix F). Students broke into groups of three and assumed the role of interviewer, patient, or observer. The interviewer was tasked with performing the HEEADSSS assessment on the patient. The observer was provided a feedback rubric to fill out while watching the encounter (Appendix G). After completion of each patient scenario (each lasting approximately 5–7 minutes), students received feedback from the peer observer and then rotated roles for the next script. We reserved approximately 10 minutes at the end of the session for a debrief, where students shared their experiences during the encounters.

### After-Hours Session

To further increase opportunities for students to interact with real children, we designed an optional “after-hours” session at our institution’s Center for Experiential Learning. This facility consists of traditional examination rooms with tools for students to learn and practice clinical skills. Faculty, fellows, and residents were invited to bring their children in for student examination practice and/or lead a physical examination learning session. We sent an email invitation to the students; RSVPs were requested to plan for the number of children needed. We ran eight rooms during this one-hour event during the final week (week 6) of the course (cloud, Fig. 1). Each room consisted of one facilitator, two to three students, and one to two patients. At our session, we had children ages 6 months to 10 years old. Facilitators were provided with the same infant/newborn and child examination checklists that were utilized during bedside teaching sessions (Appendices C, D). Facilitators demonstrated each maneuver, and then students took turns practicing on the child while the facilitator gave feedback. Students were also encouraged to interact with the children outside of performing the maneuvers.

### Assessment Strategies

We developed an online instrument to assess our intervention. We administered an anonymous REDCap survey to assess student confidence before and after participating in the curriculum [29, 30]. The pre-participation survey consisted of six 4-point Likert scale questions assessing confidence in performing a physical examination on infants and children, identifying normal findings on examination, and knowing the components of and performing a HEEADSSS assessment (1 = “strongly agree” and 4 = “strongly disagree” in feeling confident in the various domains) (Appendix H). The post-participation survey included these same questions, in addition to a question assessing participation in the optional after-hours session, a question about learning objectives being met, and two qualitative questions asking about strengths and areas for improvement (Appendix I), which two authors (L.C.P. and L.S.S.) inductively coded for themes. The survey questions were developed following synthesis of literature review by two authors (L.C.P. and L.S.S.) and validation by the course directors and the Dean of Medical Student Affairs.

### Statistical Analysis

Results of Likert questions were reported as median (IQR). We compared students’ pre- and post-participation assessments using Wilcoxon rank sum tests because observations were not paired. All analyses were performed using Stata version 14.2 (StataCorp LP, College Station, TX).

## Results

### Participants

A total of 97 first-year medical students participated in the curriculum. Pre-participation, 56 students (58%) completed the survey. Post-participation, 32 students (30%) completed the survey. Responses were not paired.

### Outcomes Results

After participating in our curriculum, students scored higher on confidence in all domains (Table 1). They felt more confident identifying normal infant (median [interquartile range]; 2 [2, 2] vs 4 [3, 4]; *p* < 0.001) and child (2 [2, 2] vs 3 [3, 4]; *p* < 0.001) examination findings, and HEEADSSS assessment components 2 [1.5,2] vs 4 [3, 4]; *p* < 0.001). They had significantly higher scores on confidence performing infant (2 [2,2.5] vs 4 [3, 4]; *p* < 0.001), child (2 [2, 2] vs 3 [3, 4]; *p* < 0.001), and HEEADSSS assessment (2 [2, 3] vs 4 [3, 4]; *p* < 0.001).

**Table 1:** Student-reported confidence across pediatric examination domains

Topic (“I feel confident…”)	Pre^a^ (*N* = 56)	Post^a^ (*N* = 32)	*p*
Examining newborn/infant	4 (3,4)	2 (2,2.5)	< 0.001
Identifying normal newborn/infant findings	4 (3,4)	2 (2,2)	< 0.001
Examining child	3 (3,4)	2 (2,2)	< 0.001
Identifying normal child findings	3 (3,4)	2 (2,2)	< 0.001
Knowledge of HEEADSSS components	4 (3,4)	2 (1.5,2)	< 0.001
Performing HEEADSSS assessment	4 (3,4)	2 (2,3)	< 0.001

Increased confidence with statistical significance across all domains

^a^Rated on a 4-point scale (1 = *Strongly agree*, 4 = *Strongly disagree*)

We did not find a significant difference in confidence outcomes in the students who had participated in the optional after-hours session versus those who had not.

The datasets generated during and/or analyzed during the current study are available from the corresponding author on reasonable request.

### Student Evaluation of Curriculum

Through inductive coding of the qualitative feedback, the authors identified several major themes: students enjoyed practicing with manikins and real patients, receiving individual guidance, and working closely with residents (Table 2).

**Table 2:** Student evaluation of curriculum

Theme	Direct Quotations from Students
Students enjoyed practicing with manikins and real patients	“The infant exam on manikins as well as the bedside teaching sessions were really informative.” “The infant exam practice was a good way to get us to be more comfortable around the procedure, doing it [for the first time] on a real infant would be intimidating. Bedside teaching sessions were super helpful because that was where we could apply what we learned!”
Students appreciated receiving individual guidance	“Someone guiding us was so useful.”
	“The one-on-one feedback was very helpful.”
Students valued working closely with residents	“Hands on practice in small groups with people in the field was super helpful.”
	“The residents did a great job walking us through each step and they were very patient”
Students at the optional after-hours session perceived added benefits from participation	“The after-hours session was fantastic!” “I really enjoyed the optional session. I got much more one-on-one feedback compared to other sessions.” “It was helpful to see how real children behave in exams during the after-hours session.” “After hours was great to get a chance to examine older kids.”

## Discussion

Our novel preclinical pediatrics curriculum provides a suggested approach to enhancing medical students’ exposure to this unique patient population and bridging the gap between the preclinical and clinical years. Our results showed that participation in the curriculum led to increased student confidence examining and assessing pediatric patients, and the curriculum was well-received by the participants.

A systematic review of the literature by Surmon et al. identified several themes related to student perceptions of preparedness (vs ill-preparedness) for clerkship, including competence, disconnection, and curriculum content [31]. A student’s perceived lack of competence in knowledge and skills at the start of clerkship can cause stress and negatively impact their learning experience [31]. Disconnection between the content of preclinical and clinical years led to difficulties with transitioning to clerkship, as noted by both clerkship directors and students [31]. Students also noted that disconnection led to decreased levels of motivation [31]. Content of preclinical clinical skills curricula was cited as a positive influence on preparedness, specifically as it pertains to the inclusion of early contact with patients and bedside teaching for contextualization of information [31]. The systematic review by Mookherjee et al. similarly identified students’ desire for early exposure to real pediatric patients [12]. Our curriculum successfully unified these themes; and thus, we hypothesize that it also lends itself to the downstream effect of increased preparedness for pediatrics clerkship. Laitman specifically studied this outcome in addition to student confidence in a similar preclinical pediatrics curriculum; however, participation in their curriculum was optional [8]. We would argue that since all students will rotate through a pediatrics rotation, mandatory participation in a pediatrics preclinical curriculum with well-demonstrated benefits is essential.

While the focus of our study was physical examination skills, another inherent skill students developed through participation in the curriculum was the ability to interact and communicate with pediatric patients, which can be one of the most daunting aspects of pediatric medicine [1]. Although we did not observe a statistically significant difference in confidence in the students who participated in the after-hours session and those who did not, students who participated in the session remarked that interacting with children in a simulated clinical setting helped them learn how to redirect and engage the children during the encounter. In another study that immersed preclinical students into a pediatric subspecialty clinic, similar findings of increased confidence with communication and empathy were reported [9]. Being able to communicate at a developmentally appropriate level is arguably one of the most important skills to possess in order to effectively examine a pediatric patient, and future directions may include the development of curriculum aimed at teaching communication skills in the context of developmental milestones.

A preclinical pediatrics curriculum offers an additional benefit of alleviating the pressure pediatric clerkship directors may face when attempting to maximize clinical skills education in an already time-limited clerkship. Our small-group, near-peer approach required minimal resources and faculty involvement and greatly increased medical students’ active learning opportunities prior to clerkship.

An important lesson learned during the implementation phase of the curriculum was the importance of standardization of the various experiences, which was frequently mentioned during student and facilitator feedback. We identified two areas for improvement in standardization. From a facilitator standpoint, it would have been beneficial to have an in-person physical examination training session prior to the start of the course to ensure that all facilitators were proficient in performing the required maneuvers. From a patient standpoint, identifying patients of a specific age range for bedside teaching sessions (for example, 1–2 months and 6–7 years old) would have been helpful due to variations in approaches to the pediatric examination by developmental age. Standardization of the patient experience across sessions was difficult due to a constantly fluctuating inpatient census.

There were several limitations to our study. First, we acknowledge the low response rate on our surveys, especially post-participation. While students were encouraged to fill out the pre-participation survey prior to the first in-person session, the minority did. We provided an additional opportunity to complete the survey during the first 5 min of the first session, but because survey participation was not required, only about half of the students responded. There was no large group session at the end of the course for students to be prompted to fill out the post-participation survey. Instead, students again received an email invitation to complete the survey with one subsequent reminder email. We believe that having students complete the post-participation survey at the last in-person session or providing incentives for participation would be helpful in achieving higher response rates. With the low response rates, we must consider the effect of response bias, as perhaps only students who found the session to be beneficial responded. Alternatively, the students who responded may have been mainly students who were interested in pursuing pediatrics. Due to the inability to withhold an entire component of the course curriculum to establish a control group, we were unable to compare the impact of our curriculum on those who participated versus a group who did not. There are also some limitations to transferability of our curriculum, as it requires significant resident involvement to facilitate the various sessions, as well as an easily accessible academic medical center for bedside teaching sessions, which may not be feasible for every medical school.

We are currently performing a retrospective study on student confidence after participation in our curriculum now that they have begun their pediatrics clerkship. Data collection is ongoing. Future studies should include analyzing the relationship between student confidence and competence in these skills after participation through a clinical skills assessment and clerkship outcomes before and after curriculum implementation. We would also be interested in studying the effects of teaching these skills on the near-peer teacher. Future directions should include the incorporation of pediatric-specific content and hands-on practice into traditional pre-clerkship clinical skills courses. In this way, students can enter clerkship feeling prepared, build upon an already established foundation of knowledge, and master clinical skills essential to pediatrics.

## Supplementary Information

Below is the link to the electronic supplementary material.

A. Newborn Physical Exam Video: A nurse practitioner demonstrates her head-to-toe approach of the newborn physical examination (MP4 484993 KB)

B. Newborn Exam Didactic: A PowerPoint presentation providing an overview of the newborn/infant physical examination and important normal vs abnormal findings (PDF 270 KB)

C. Newborn Exam Checklist: A checklist encompassing key components of the newborn/infant physical examination (PDF 143 KB)

D. Child Exam Checklist: A checklist encompassing key components of the child physical examination (PDF 135 KB)

E. HEEADSSS Assessment Didactic: A PowerPoint presentation reviewing the components of the HEEADSSS assessment and suggested approaches to asking sensitive questions. This presentation is given prior to the role play activity (PDF 199 KB)

F. Patient Scripts: A set of three patient scripts with HEEADSSS-relevant information for use during the role play activity (PDF 223 KB)

G. Observer Feedback Tool: A rubric to use during the HEEADSSS role play activity. Student not engaged in “doctor” or “patient” role should fill out based on “doctor” student’s interview skills (PDF 167 KB)

H. Pre-Intervention Survey: This REDCap survey consists of 5 Likert-style confidence questions was sent to students prior to participation in the curriculum, asking them to rank their confidence in pediatrics-relevant examination skills (PDF 144 KB)

I. Post-Intervention Survey: This REDCap survey consists of the same Likert-style confidence questions as the pre-intervention survey, in addition to 5 additional questions on qualitative feedback and participation in the optional after-hours activity. This was sent to students after participation in the curriculum (PDF 157 KB)

## Declarations

### Disclaimer

The sponsors and funders had no role in the design and conduct of the study or in the collection, analysis, and interpretation of the data, or in the preparation, review, or approval of the manuscript.
