# Supplementary material for: A Novel Pediatric Clinical Skills Curriculum to Prepare Medical Students for Pediatrics Clerkship
Source: Med Sci Educ. 2024 Nov 13;35(1):343–50. doi: 10.1007/s40670-024-02191-w (PMC11933490; doi:10.1007/s40670-024-02191-w)
Supplement: Supplementary file 2 — B. Newborn Exam Didactic: A PowerPoint presentation providing an overview of the newborn/infant physical examination and important normal vs abnormal findings (PDF 270 KB) [file 40670_2024_2191_MOESM2_ESM.pdf]

A Novel Pediatric Clinical Skills Curriculum to Prepare Medical Students for Pediatrics Clerkship

Lindsay Podraza, MD[1]; Lauren S. Starnes, MD, MEd[2]; Joseph R. Starnes, MD, MPH[3], Anuj Patel, MD[4]; Rachel K.P. Apple, MD, MPH[5]

Contributor: Lauren Presley, MSN APRN, CPNP-PC[6]

[1] Pediatric Resident, Monroe Carell Jr. Children's Hospital at Vanderbilt, Nashville, TN, USA. ORCID 0000-0002-4926-0001

[2] Pediatric Hospital Medicine Fellow, Monroe Carell Jr. Children's Hospital at Vanderbilt, Nashville, TN, USA. ORCID 0000-0001-7075-9774

[3] Pediatric Cardiology Fellow, Monroe Carell Jr. Children's Hospital at Vanderbilt, Nashville, TN, USA. ORCID 0000-0001-7954-5385

[4] Assistant Professor of Pediatrics, Monroe Carell Jr. Children's Hospital at Vanderbilt, Nashville, TN, USA

[5] Associate Professor of Internal Medicine and Pediatrics, Vanderbilt University Medical Center, Nashville, TN, USA

[6] Pediatric Nurse Practitioner, Newborn Nursery, Vanderbilt University Medical Center, Nashville, TN, USA

Corresponding author: Lindsay Podraza, lindsaypodraza.md@gmail.com

NEWBORN EXAM

Objectives

At the end of this session, students will feel confident:

- Systematically examining a healthy newborn/infant
- Recognizing normal newborn/infant physical examination findings

GENERAL

- Wear gloves!
- Baby should be quiet when auscultating heart/lungs
    - Calming maneuvers

HEART

- Auscultate 4 areas
  - Left upper sternal border
  - Left lower sternal border
  - Apex
  - Right upper sternal border
- Auscultate on front, back, axillae
- Tip: Transitional murmurs can sometimes be heard better with bell side!

LUNGS

- Auscultate upper and lower lobes bilaterally on anterior and posterior sides
- Determine work of breathing
  - Nasal flaring
  - Grunting
  - Retractions
  - Head bobbing

HEAD

- Quality of fontanelles
  - Flat
  - Full
  - Sunken
  - Bulging
  - Tense
  - If any of the above concerns, assess while lying flat and sitting up
- Head shape
  - cone head = molding
  - cephalohematoma
  - subgaleal
  - caput
- Sutures (overriding?)
- lacerations/abrasions (forceps assisted deliveries)

EARS

- Positioning
- Appearance
    - Abnormalities: Ear tags, pits, etc
- No need for otoscope exam in this age group!

EYES

- Red reflex
  - Maneuvers
- Conjunctiva → any hemorrhage?
- EOM not expected to be in sync yet

NOSE

- Patency
- Congestion can be normal after being in amniotic fluid

MOUTH

- Palate - cleft?
- Tongue tie? (consider if poor suck, difficulty feeding)
- Neuro exam: suck
- Rooting reflex: tickle side of mouth and head should turn that way

CLAVICLE

- Palpate for step offs (birth trauma - big babies (IDM); shoulder dystocia) = clavicular fracture

ABDOMEN

- Auscultation of bowel sounds
- Palpate liver edge
- Palpate for masses (cystic kidneys, for example)

UMBILICAL CORD

- Normal = 2 arteries + 1 vein

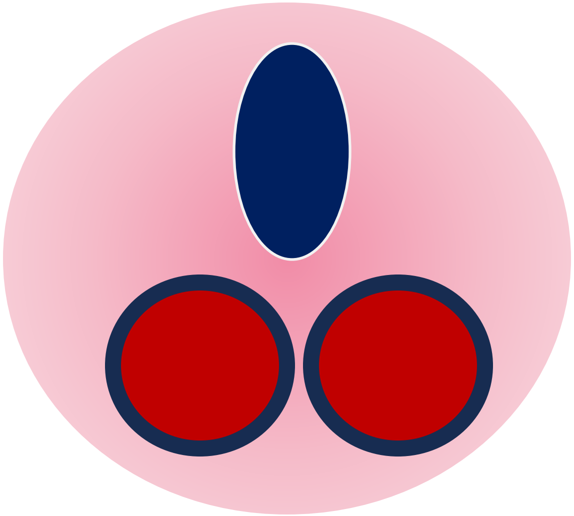

DIAPER AREA

- Pulses: femoral
- Patency of anus
- Males:
  - Scrotum/testes: descended bilaterally?
    - Technique: should use pointer finger to block inguinal canal while doing downward milking motion - not pinching upward
  - Penile shaft: hypo-/epispadias? Torsion?
- Females:
  - Labial fusion
  - Discharge
  - Tags
  - Clitoromegaly

HIPS

- Barlow (BA-back)
  - Feel a shift backwards if hip dislocates
- Ortolani (O- out)
  - Feel a clunk/pop when hip goes back into place (if previously dislocated)
- Tip: Positioning for maneuver -
  - Thumb on inside of knee
  - 3rd/4th fingers over hip joints
  - Pelvis in neutral position
  - One side at a time

BACK

- Palpate spine
  - Curvature?
  - Masses?
- Sacral dimple/tuft?
  - Visible base?

SKIN

- Inspect entire body in the light!
- Rashes
- Birthmarks, hemangiomas, bruising, congenital dermal melanocytosis, milia, E. tox
- Jaundice

NEURO

- Suck
- Moro (startle)
- Plantar grasp
- Tone

EXTREMITIES

- Appropriate number/appearance of fingers and toes? Webbed digits? Polydactyly?
- Cap refill - finger with arm at heart level

Putting it all together…

- Practice head-to-toe exam with your small group!
- Practice swaddling (which you should always do before leaving the room!)

References

14. Biancaniello T. Innocent Murmurs. *Circulation*. 2005;111(3). doi:10.1161/01.cir.0000153388.41229.cb

15. Hermansen CL, Lorah KN. Respiratory distress in the newborn. *Am Fam Physician*. 2007;76(7):987-994.

16. Aby J. Photo Gallery. Newborn Nursery Photo Gallery. Accessed January 24, 2024. https://med.stanford.edu/newborns/professional-education/photo-gallery/ears.

17. AMERICAN ACADEMY OF PEDIATRICS, Section on Ophthalmology, AMERICAN ASSOCIATION FOR PEDIATRIC OPHTHALMOLOGY AND STRABISMUS, AMERICAN ACADEMY OF OPHTHALMOLOGY, AMERICAN ASSOCIATION OF CERTIFIED ORTHOPTISTS; Red Reflex Examination in Neonates, Infants, and Children. *Pediatrics* December 2008; 122 (6): 1401–1404. 10.1542/peds.2008-2624

18. Horwood AM. Typical and Atypical Development of Ocular Alignment and Binocular Vision in Infants–The Background. Knights Templar Eye Foundation Pediatric Ophthalmology Education Center. June 10, 2019. Accessed January 24, 2024. https://www.aao.org/education/disease-review/typical-atypical-development-of-ocular-alignment-b

19. Newborn Nursery. Barlow and Ortalani Manuevers. Newborn Nursery. Accessed January 24, 2024. https://med.stanford.edu/newborns/clinical-rotations/residents/residents-newborn-exam/barlow-and-ortalani-manuevers.html.

https://rise.articulate.com/share/zbhfUDDWdCT_sMbI5P2rcJjJAG9xtBom#/lessons/bPUlINa4YbCkJXD5Ov1ygEy9QRcpOAUk. Accessed January 24, 2023.
