# Supplementary material for: A Novel Pediatric Clinical Skills Curriculum to Prepare Medical Students for Pediatrics Clerkship
Source: Med Sci Educ. 2024 Nov 13;35(1):343–50. doi: 10.1007/s40670-024-02191-w (PMC11933490; doi:10.1007/s40670-024-02191-w)
Supplement: Supplementary file 3 — C. Newborn Exam Checklist: A checklist encompassing key components of the newborn/infant physical examination (PDF 143 KB) [file 40670_2024_2191_MOESM3_ESM.pdf]

A Novel Pediatric Clinical Skills Curriculum to Prepare Medical Students for Pediatrics Clerkship

Lindsay Podraza, MD[1]; Lauren S. Starnes, MD, MEd[2]; Joseph R. Starnes, MD, MPH[3], Anuj Patel, MD[4]; Rachel K.P. Apple, MD, MPH[5]
Contributor: Lauren Presley, MSN APRN, CPNP-PC[6]

[1] Pediatric Resident, Monroe Carell Jr. Children's Hospital at Vanderbilt, Nashville, TN, USA. ORCID 0000-0002-4926-0001
[2] Pediatric Hospital Medicine Fellow, Monroe Carell Jr. Children's Hospital at Vanderbilt, Nashville, TN, USA. ORCID 0000-0001-7075-9774
[3] Pediatric Cardiology Fellow, Monroe Carell Jr. Children's Hospital at Vanderbilt, Nashville, TN, USA. ORCID 0000-0001-7954-5385
[4] Assistant Professor of Pediatrics, Monroe Carell Jr. Children's Hospital at Vanderbilt, Nashville, TN, USA
[5] Associate Professor of Internal Medicine and Pediatrics, Vanderbilt University Medical Center, Nashville, TN, USA
[6] Pediatric Nurse Practitioner, Newborn Nursery, Vanderbilt University Medical Center, Nashville, TN, USA

Corresponding author: Lindsay Podraza, lindsaypodraza.md@gmail.com

NEWBORN EXAM CHECKLIST:

Objectives:

1. Increase medical student confidence in systemically performing a newborn/infant exam.

2. Increase medical student confidence in recognizing normal newborn/infant exam findings.

GENERAL:

- Listens to heart/lungs when baby is quiet
- Calming maneuvers
- Wear gloves

HEART:

- LUSB, LLSB, apex, RUSB (bell side too)
- Peripheral pulmonic pulmonary stenosis - a normal murmur (heard at LUSB, radiates to back/axilla; due to turbulent flow in collaterals off of pulmonary artery; should go away with time)
    - Transitional murmurs can be heard better with bell sometimes

LUNGS:

- Auscultate in upper and lower lobes on anterior side bilaterally (can do posterior too)
- Signs of increased work of breathing:
    - Grunting
    - Nasal flaring
    - Retractions
    - Head bobbing
    - Tracheal tugging

EYES:

- Red reflex (must actually open the eye)
    - Demonstrate lights off technique
    - Picking baby up
    - Have someone help open eye for you
- Conjunctivae (looking for hemorrhage)
- EOM not expected to be in sync yet

HEAD:

- Fontanelles (anterior + posterior): open, full/tense/sunken/flat (if concerns, assess lying down and sitting up)

    - Normal = flat
    - Sunken = dehydration
    - Full/tense/bulging = concern for meningitis or increased ICP
- Head shape
    - cone head - molding
    - overriding sutures
    - cephalohematoma - confined by suture lines, on right or left side of head with well defined borders, feels dense, persist for weeks or months, can calcify;
    - subgaleal
    - caput - present at birth, posterior aspect of head, boggy; usually resolves in a few days
- Lacerations, abrasions

EARS:
- NO otoscope exam
- Shape
- Positioning
- Ear tags? Ear pits?

NOSE: obligate nose breathers
- Nares patent
- Congestion can be normal after inhaling amniotic fluid

MOUTH:
- Wear gloves
- Feel palate with pinky finger, nail side down
- Tongue tie? (esp if poor suck, difficulty breastfeeding)
- Suck
- Ebstein pearls
- Rooting reflex: tickle side of mouth and should come towards finger

NECK:
- Lift up on chin, make sure there are no masses or anything else concerning (thyroglossal duct cyst)

CLAVICLES:
- Palpate along both sides. Looking for step off (clavicular fracture) esp in big babies/shoulder dystocia baby

CHEST:

- Breast buds (normal in boys/girls)
- Prominent xiphoid process in some babies

ABDOMEN:

- Inspect
- Auscultate
- Palplate
    - Masses
    - Liver edge?
    - Soft, non-distended

UMBILICAL CORD (will not be able to demonstrate on manikin):

- 2 arteries + 1 vein

DIAPER AREA:

- Femoral pulses (present? symmetric?) - abduct leg out and follow femur in
- Males:
    - Scrotum, testes (descended bilat? Palpate with index finger blocking inguinal canal, milking downward motion), hydrocele?, circ?, hypo-/epispadias?, torsion? Chordee?
- Females:
    - Labia majora should completely (or almost completely) cover minora
    - Vaginal discharge can be present (related to maternal hormones). Can be bloody, still normal. Resolves in 1-2 weeks after birth
- Anus: patent? (has the infant stooled?)

HIP EXAM:

- Barlow (BA-back)/Ortolani (O - out): thumb on inside of knee. Third and fourth fingers over hip joint. Diaper off, one side at a time. Pelvis in neutral position. Have to apply pressure.
    - Barlow - feel shift back
    - Ortolani - may hear/feel clunk or pop when hip goes back into place

BACK:

- Palpate along spine
    - Curvature? Masses?

- Sacral dimple? Visualize base?
- Hair tuft?

SKIN:
- Hemangioma
- Congenital dermal melanocytosis
- Milia
- bruising/abrasions from birth trauma?
- Erythema toxicum
- Jaundice

NEURO:
- Startle (moro reflex) - symmetric, cry
- Plantar grasp
- Suck
- Tone

EXTREMITIES:
- Normal # fingers/toes? Webbed digits?
