# Supplementary material for: A Novel Pediatric Clinical Skills Curriculum to Prepare Medical Students for Pediatrics Clerkship
Source: Med Sci Educ. 2024 Nov 13;35(1):343–50. doi: 10.1007/s40670-024-02191-w (PMC11933490; doi:10.1007/s40670-024-02191-w)
Supplement: Supplementary file 4 — D. Child Exam Checklist: A checklist encompassing key components of the child physical examination (PDF 135 KB) [file 40670_2024_2191_MOESM4_ESM.pdf]

A Novel Pediatric Clinical Skills Curriculum to Prepare Medical Students for Pediatrics Clerkship

Lindsay Podraza, MD[1]; Lauren S. Starnes, MD, MEd[2]; Joseph R. Starnes, MD, MPH[3], Anuj Patel, MD[4]; Rachel K.P. Apple, MD, MPH[5]
Contributor: Lauren Presley, MSN APRN, CPNP-PC[6]

[1] Pediatric Resident, Monroe Carell Jr. Children's Hospital at Vanderbilt, Nashville, TN, USA. ORCID 0000-0002-4926-0001
[2] Pediatric Hospital Medicine Fellow, Monroe Carell Jr. Children's Hospital at Vanderbilt, Nashville, TN, USA. ORCID 0000-0001-7075-9774
[3] Pediatric Cardiology Fellow, Monroe Carell Jr. Children's Hospital at Vanderbilt, Nashville, TN, USA. ORCID 0000-0001-7954-5385
[4] Assistant Professor of Pediatrics, Monroe Carell Jr. Children's Hospital at Vanderbilt, Nashville, TN, USA
[5] Associate Professor of Internal Medicine and Pediatrics, Vanderbilt University Medical Center, Nashville, TN, USA
[6] Pediatric Nurse Practitioner, Newborn Nursery, Vanderbilt University Medical Center, Nashville, TN, USA

Corresponding author: Lindsay Podraza, lindsaypodraza.md@gmail.com

CHILD EXAM CHECKLIST

Objectives:

1. **Increase medical student confidence in systemically performing a head-to-toe exam of a child.**
2. **Increase medical student confidence in identifying normal child exam findings.**

Before beginning, acknowledge the differences and adaptations necessary to examine children (vs adults):

- Patient cooperation is often difficult to win!
- Include parents/caregivers
- Engage in age-appropriate conversation
- Make a game out of it!
- Give breaks
- Do NOT ask permission if you are going to perform the exam anyways
- End with the most distressing maneuvers (ears, throat)

GENERAL:

- Overall appearance (well- vs ill- or toxic-appearing; active vs lethargic)
- Behavior
- Growth (appropriate for age?)
- Vital signs

SKIN:

- Temperature to the touch (cool, cold, warm)
- Color (pale, pink, mottled)
- Lesions
- Rashes
- Scars
- Erythema/drainage/wounds?

HEENT:

- Mucous membranes: dry vs moist
- Head
    - Shape (symmetric?)
    - Signs of trauma
    - Facies (dysmorphisms)
- Eyes
    - Cornea

- Iris
- Conjunctiva
- Sclera
- Gaze (conjugate, symmetric)
- Corneal light reflex test
- Ears (save until end to avoid patient fussiness)
  - External ear
  - Ear canal
  - Tympanic membranes (landmarks: review picture attached as this is difficult to demonstrate on real patient)
  - Gross hearing ability
- Nose
  - Mucous membrane appearance (pale/boggy, moist vs dry, color)
  - Congestion or rhinorrhea
- Mouth/Pharynx
  - Lips/perioral area
  - Tongue
  - Tonsils
  - Uvula
  - Palate
  - Teeth

NECK:
- Lymph nodes
- ROM

LUNGS:
- 3 spots anteriorly and posteriorly on each lung (don't forget RML!)
  - Equal air entry bilaterally? Clear (vs crackles, coarse, wheezing)
- Inspiratory:expiratory ratio should be 1:2-1:3 (expiration takes 2-3x as long as inspiration) → prolonged in asthma/obstruction
- Work of breathing (nasal flaring, grunting, head bobbing, retractions, tracheal tugging)
- Palpation
- Percussion

HEART:
- Areas to listen: aortic, pulmonic, tricuspid, mitral (murmurs, rubs, gallops)

ABDOMEN:
- Inspect
- Auscultate
- Palpate (superficial then deep)

    - Liver: scratch test, start low and work way upwards
    - Spleen maneuver: two hands, instruct pt to take deep breath

MSK:
- Range of motion
- Edema

NEURO/DEVELOPMENT:
- Cranial nerves
- Sensory (eyes closed)
- Gait, coordination
- Strength
- Deep tendon reflexes
- Cerebellum tests: FNF, rapid alternating movements (if age appropriate)
- Talk/interact with patient to assess gross development

GENITOURINARY: Will not perform
- Very important: Emphasize to students that you should explain that the GU exam is safe because you are a doctor; you would not expect strangers or anyone other than a trusted parent/caregiver to be looking/touching this area
