# Supplementary material for: A Novel Pediatric Clinical Skills Curriculum to Prepare Medical Students for Pediatrics Clerkship
Source: Med Sci Educ. 2024 Nov 13;35(1):343–50. doi: 10.1007/s40670-024-02191-w (PMC11933490; doi:10.1007/s40670-024-02191-w)
Supplement: Supplementary file 5 — E. HEEADSSS Assessment Didactic: A PowerPoint presentation reviewing the components of the HEEADSSS assessment and suggested approaches to asking sensitive questions. This presentation is given prior to the role play activity (PDF 199 KB) [file 40670_2024_2191_MOESM5_ESM.pdf]

A Novel Pediatric Clinical Skills Curriculum to Prepare Medical Students for Pediatrics Clerkship

Lindsay Podraza, MD[1]; Lauren S. Starnes, MD, MEd[2]; Joseph R. Starnes, MD, MPH[3], Anuj Patel, MD[4]; Rachel K.P. Apple, MD, MPH[5]

Contributor: Lauren Presley, MSN APRN, CPNP-PC[6]

[1] Pediatric Resident, Monroe Carell Jr. Children's Hospital at Vanderbilt, Nashville, TN, USA. ORCID 0000-0002-4926-0001

[2] Pediatric Hospital Medicine Fellow, Monroe Carell Jr. Children's Hospital at Vanderbilt, Nashville, TN, USA. ORCID 0000-0001-7075-9774

[3] Pediatric Cardiology Fellow, Monroe Carell Jr. Children's Hospital at Vanderbilt, Nashville, TN, USA. ORCID 0000-0001-7954-5385

[4] Assistant Professor of Pediatrics, Monroe Carell Jr. Children's Hospital at Vanderbilt, Nashville, TN, USA

[5] Associate Professor of Internal Medicine and Pediatrics, Vanderbilt University Medical Center, Nashville, TN, USA

[6] Pediatric Nurse Practitioner, Newborn Nursery, Vanderbilt University Medical Center, Nashville, TN, USA

Corresponding author: Lindsay Podraza, lindsaypodraza.md@gmail.com

Interviewing the Adolescent Patient:

The HEEADSSS Assessment

Objective:

At the end of this session, students will feel confident identifying components included in a HEEADSSS assessment and performing the HEEADSSS assessment.

What might be some unique aspects of history-taking in the adolescent population?

HEEADSSS

Home/Environment

Education

Eating/Exercise

Activities/Employment

Drugs/Substances

Suicidality/Depression

Sexuality

Safety

Why is this important?

Leading causes of mortality in this age group:

1) **Accidents (unintentional injuries) → ½ of all teen deaths come from this category**
   a) **MVCs are #1 cause**
2) **Homicide**
3) **Suicide**
4) Cancer
5) Heart disease

Risky behavior & environmental/social factors are important health determinants!

Before you begin:

- Ask everyone else to step out of room (parents and guardians included)
  - "Since you are getting close to becoming a legal adult, we will have you practice answering some questions without the help of your parent/guardian. We want you to be able to speak openly and honestly with doctors in case you ended up in an emergency room or doctor's office alone in the future."
  - "This will only take a few minutes."
- Statement of confidentiality
  - Limitations:
    - Suspected physical/sexual abuse
    - Suspected risk of suicide/homicide
    - Required reporting of certain diseases (chlamydia, GC, HIV, TB)
- Helpful tip: Begin with developmentally-appropriate "opener" questions, then move to focused questions.

H: Home

Opener example: "How are things going at home?"

Follow up:

- Where do you live? (House, apartment, condo, etc. / city or area)
- How long have you lived there?
- Who lives at home with you? Do you get along with them?
- Does anyone come to stay at your home sometimes? (Visiting family members, parents' friends/partners)
- Do you feel safe at home? How about in your neighborhood?
- Are there any guns in your home? *If yes, Do you know where they are stored? Are they locked up? Do you know how to get access to them?*

E: Education

Opener example: “Are you in school right now?”

Follow up:

- What grade are you in this year?
- What school do you go to? Have you always gone to that school?
- What kind of grades do you get?
- Do you feel safe at school?
- Do you have a friend group?
- Have you missed any school days this year?
- Have you ever been suspended/expelled before?
- What do you want to do when you are done with school?

Eating/Exercise

- Do you feel comfortable with the way your body looks?
  - *If no/unsure: Have you ever dieted before? Have you ever restricted the amount of food or calories you allow yourself to eat? Do you ever make yourself throw up after you eat to lose weight? Have you ever used diet pills or laxatives to help lose weight?*
- Do you exercise?

A: Activities/employment

Opener: What do you like to do for fun?

Follow up:

- Do you have a group of friends? *If yes, what do you and your friends do when you hang out together?*
    - Helpful tip: Do not assume that every teen has friends. This can be triggering/demolish rapport if assumed.
- Are you in any clubs or after-school activities? Sports?
- Do you have a job? *If yes, where? How many hours per week?*

D: Drugs

Helpful tip: Start with what their friends do/don’t do to help build rapport/trust

Opener: Do any of your friends smoke or drink? What do they smoke (cigs, MJ, vape, etc.). Do you know anyone who smokes or drinks?

Follow up:

- Have you ever tried? *If yes, what have you tried? (Recommend naming concrete examples: MJ, alcohol (hard liquor vs beer or wine), LSD, mushrooms, cocaine, heroin, cigarettes. Have you ever used needles to inject drugs?). How often have you used these things? Where do you get them from?*

D: Drugs (continued)

CRAFFT questions (substance abuse screening)

C: Have you ever ridden in a **CAR** driven by someone (including yourself) who was "high" or had been drinking/using drugs?

R: Do you ever use alcohol/drugs to **RELAX**, fit in, or feel better about yourself?

A: Do you ever use drugs/alcohol when you are **ALONE**?

F: Do you ever **FORGET** things you did while using?

F: Do your **FAMILY** or **FRIENDS** tell you that you should cut down on your drug/alcohol use?

T: Have you ever gotten into **TROUBLE** while using drugs/alcohol?

2+ yes responses = serious problem

S: Suicidality/(Depression)

Helpful tip: Give a warning shot before delivering these questions.

Example: “The next questions I’m going to ask you can be uncomfortable, triggering, or awkward, but I ask all my patients these questions because I care about their safety.”

- Do you have any concerns about your mood?
- SIG E CAPS
  - Sleep
  - Interest (anhedonia)
  - Guilt/worthlessness
  - Energy
  - Concentration
  - Appetite
  - Psychomotor slowing
  - Suicidality

- Have you ever felt so sad that you considered hurting yourself? Killing yourself?
    - These are two separate things! Must ask both!
    - Have you ever tried to hurt/kill yourself? Tell me more (determine mechanism- cutting, overdose, hanging, etc.)
    - Are you currently having thoughts of wanting to hurt/kill yourself? *If yes to either, do you have a plan?*
    - Do you know how to seek help if you feel that you cannot keep yourself safe? (Trusted family/friend, suicide hotline, etc.

S: Sexuality

Warning shot: “Now I’m going to ask you some questions about relationships. Again, these can get awkward, but I just want to make sure I’m thinking about your health and safety from all perspectives.”

- Are you interested in boys/girls/both/neither/unsure?
  - Important for understanding risk of sexually transmitted infections, pregnancy, etc.
- Are you in a relationship?
  - If yes: Is your partner male/female/other? Do you feel safe in that relationship?
  - If no: Have you ever been in a relationship? What was that like?
- Are you currently sexually active? Have you ever been sexually active? How many partners? Male or female?
  - Helpful tip: Define what you mean by “sexually active.”

- Do you masturbate? If necessary (for example: if concerned for a related infection, can dive into details).
- Have you ever been pregnant?
- Do you use protection? What kind?
- Have you ever had an STD? Have you ever been tested?
- Have you ever traded sex for money, drugs, alcohol, or other things?

Safety

Opener: Do you ever feel that your decisions put you at risk of being unsafe?

Follow up:

- Do you always wear a seatbelt in the car?
- Have you met up with somebody you met online before?
- Are you exposed to violence at home or at school?
- Do you text and drive?
- Have you ever been in trouble with the police?
- Have you ever been in a physical fight? When was the last time?

Closing the interview

- Thank patient for being open and vulnerable
- Reassure about confidentiality and address limitations if needed
- Remind patient that healthcare settings are safe places to discuss these matters/seek help
- Ask if they have any questions regarding topics discussed
- Provide next steps/follow-up

Time to practice!

References

Katzenellenbogen R. HEADSS: The "Review of Systems" for adolescents. *AMA Journal of Ethics*. 2005;7(3). doi:10.1001/virtualmentor.2005.7.3.cprl1-0503

Miniño AM. Mortality Among Teenagers Aged 12-19 Years: United States, 1999-2006. Centers for Disease Control and Prevention. https://www.cdc.gov/nchs/products/databriefs/db37.htm. Published November 6, 2015. Accessed November 18, 2022.

Ford C, English A, Sigman G. Confidential Health Care for adolescents: Position paper of the society for adolescent medicine. *Journal of Adolescent Health*. 2004;35(2):160-167. doi:10.1016/s1054-139x(04)00086-2

Knight JR, Sherritt L, Shrier LA, Harris SK, Chang G. Validity of the crafft substance abuse screening test among adolescent clinic patients. *Archives of Pediatrics & Adolescent Medicine*. 2002;156(6):607. doi:10.1001/archpedi.156.6.607
