# Supplementary material for: A Novel Pediatric Clinical Skills Curriculum to Prepare Medical Students for Pediatrics Clerkship
Source: Med Sci Educ. 2024 Nov 13;35(1):343–50. doi: 10.1007/s40670-024-02191-w (PMC11933490; doi:10.1007/s40670-024-02191-w)
Supplement: Supplementary file 6 — F. Patient Scripts: A set of three patient scripts with HEEADSSS-relevant information for use during the role play activity (PDF 223 KB) [file 40670_2024_2191_MOESM6_ESM.pdf]

A Novel Pediatric Clinical Skills Curriculum to Prepare Medical Students for Pediatrics Clerkship

Lindsay Podraza, MD[1]; Lauren S. Starnes, MD, MEd[2]; Joseph R. Starnes, MD, MPH[3], Anuj Patel, MD[4]; Rachel K.P. Apple, MD, MPH[5]

Contributor: Lauren Presley, MSN APRN, CPNP-PC[6]

[1] Pediatric Resident, Monroe Carell Jr. Children's Hospital at Vanderbilt, Nashville, TN, USA. ORCID 0000-00024926-0001

[2] Pediatric Hospital Medicine Fellow, Monroe Carell Jr. Children's Hospital at Vanderbilt, Nashville, TN, USA. ORCID 0000-0001-7075-9774

[3] Pediatric Cardiology Fellow, Monroe Carell Jr. Children's Hospital at Vanderbilt, Nashville, TN, USA. ORCID 0000-0001-7954-5385

[4] Assistant Professor of Pediatrics, Monroe Carell Jr. Children's Hospital at Vanderbilt, Nashville, TN, USA

[5] Associate Professor of Internal Medicine and Pediatrics, Vanderbilt University Medical Center, Nashville, TN, USA

[6] Pediatric Nurse Practitioner, Newborn Nursery, Vanderbilt University Medical Center, Nashville, TN, USA

Corresponding author: Lindsay Podraza, lindsaypodraza.md@gmail.com

-year-

Patient #1 Name: Daniella Romero

Patient Demographics: Patient is a 15-year-old Latina female presenting for her annual checkup prior to starting the new school year.

Patient's Chief Complaint: Annual check up

Actual Diagnosis: Depression, passive suicidal ideation, high risk behavior

HEEADSSS information:

Home:
- Live in Nashville, TN in a 3-bedroom apartment with mom, mom's boyfriend, and 18year-old brother named Luis. You have lived in this apartment your whole life. Mom's boyfriend moved in 6 months ago. Nobody else comes to stay at home.
- You feel safe at home.
- You are close with your mom. You do not get along with your mom's boyfriend. You are not close with your older brother.
- You are not aware of any guns or weapons in the home.

Education:
- Grade: Going into sophomore year at the local public high school
- School performance: First semester last year you received Straight As. Second semester your grades were Bs and Cs.
- You feel safe at school.
- You have a small friend group at school.
- Last year you missed 5 days of school during the second semester. No days missed during the first semester. Reason for missing school: "Tired and not feeling well."
- No history of suspensions/expulsions
- Future career aspirations: Special education teacher

Eating/exercise:
- Somewhat confident in the way your body looks but feel that you might be gaining weight
- No history of dieting, binging/purging/restrictive behaviors
- You do not exercise

Activities/employment:
- Employment: Cashier at local grocery store; 15 hrs/week
- Hobbies: Used to love to bake and volunteer at the local children's hospital but lately have done these things less. Reason: "I have felt less interested in doing these."
- Not involved in any school-related extracurriculars

Drugs:

- Drugs: You recently tried smoking marijuana, which you got from a friend. You've smoked twice in the last few weeks to "relax." You have never tried any other substances. Many of your friends smoke marijuana regularly.
- Alcohol: You drank for the first time two weekends ago. You had 2 shots of hard liquor and 1 beer. You drank to "relax" with friends at a party. You have never blacked out before.
- You have never done anything you regretted while using drugs/alcohol.
- Your family is not aware that you have tried marijuana/alcohol.
- You have ridden in a car with friends who smoked marijuana beforehand.

Suicidality/depression:

- Mood: "feeling down"
- Little interest in hobbies
- Feel worthless at times
- Decreased energy despite sleeping/laying in bed more
- Eating less
- Has had thoughts of killing self because "the world would be better off." Has never had a plan.
- No self-injurious behavior

Sexuality:

- Interested in men
- Not currently in a serious relationship but did start "hooking up" with a 16-year-old boy from class. This is your 1st sexual partner.
- You define "hooking up" as having casual sex
- Using condoms for contraception
- No history of STDs
- No history of pregnancy

Safety:

- Feels safe at home and at school
- Wears seatbelt in the car "most of the time"
- No history of getting in physical fights

-year-

Patient #2 Name: Thomas Smith

Patient Demographics: Patient is a 17 old Caucasian male presenting to the emergency department for pain with urination.

Patient's Chief Complaint: Pain with urination

Actual Diagnosis: Sexually transmitted disease

HEEADSSS information:

Home:
- Live in Nashville, TN in a 4-bedroom single family home with mom, dad, two sisters (aged 14 and 11). No recent moves. Nobody else comes to stay at home.
- You feel safe at home.
- You have a good relationship with all family members.
- Parents have a gun locked and stored somewhere in the home that you are not aware of.

Education:
- Grade: Senior year of high school
- School performance: A's and B's ● You feel safe at school.
- You are very popular and have a large group of friends.
- No missed school days so far.
- No history of suspensions/expulsions
- Future career aspirations: Career in finance

Eating/exercise:
- No concerns about body image
- No history of dieting, binging/purging/restrictive behaviors
- Exercise daily, also on HS football team

Activities/employment:
- Not employed
- Hobbies: Football (on school team), baseball, golf

Drugs:
- Drugs: Never used any form of drugs
- Alcohol: Drink 2-3 beers ~once a month at parties
- You have never done anything you regretted while using drugs/alcohol.
- Parents are aware of occasional drinking.
- You have never ridden in a car with somebody who was under the influence.

Suicidality/depression:
- No current or past concerns about mood
- No current or past suicidal ideation

Sexuality:
- Interested in women
- In a 6 month relationship with a female
- Sexually active (oral, vaginal sex)
- Rarely using condoms. Partner is on OCPs.
- No history of STDs

Safety:
- Feels safe at home and at school
- Wears seatbelt in the car always
- No history of getting in physical fights

-year-

Patient #3 Name: Lily Brown

Patient Demographics: Patient is a 14 old African American female presenting for a sports physical.

Patient's Chief Complaint: Sports physical

Actual Diagnosis: Well adolescent, no acute concerns

HEEADSSS information:

Home:
- Live in Antioch, TN in a 3-bedroom house with mom, dad, and 17-year-old sister. You moved from Alabama when you were 3. Nobody else comes to stay at home.
- You feel safe at home.
- You are close with your mom, dad, and sister.
- Parents have talked about a gun that is for self-defense, but you don't know where it is within your home.

Education:
- Grade: Recently started freshman year of high school
- School performance: As and Bs
- You feel safe at school.
- You have a medium-sized friend group at school.
- No history of suspensions/expulsions
- Future career aspirations: Unsure

Eating/exercise:
- Confident in how body looks
- No history of dieting, binging/purging/restrictive behaviors
- You take walks with your mom around the neighborhood when the weather is warm. You go to the YMCA 2x/week with friends to shoot hoops.

Activities/employment:
- Employment: Not employed
- Hobbies: Basketball, making Tik Tok videos
- On school basketball team

Drugs:
- Drugs: Neither you nor your friends have ever tried drugs.
- Alcohol: Neither you nor your friends have ever tried alcohol.
- You have ridden in a car with friends who were under the influence.

Suicidality/depression:

- Mood: “fine”
- Deny anhedonia
- Deny worthlessness/guilt
- Energy level normal
- Eating normal amount
- Deny any history of suicidal or homicidal ideation
- Deny hallucinations
- No self-injurious behavior

Sexuality:

- Interested in boys
- Not currently in a relationship. You have never been in a relationship.
- You have never been sexually active. You did kiss a boy 2 months ago at the playground.
- Mom has talked to you about safe sex.
- No history of STDs
- No history of pregnancy

Safety:

- Feels safe at home and at school
- Always wear seatbelt in the car
- No history of getting in physical fights
