# Supplementary material for: A Novel Pediatric Clinical Skills Curriculum to Prepare Medical Students for Pediatrics Clerkship
Source: Med Sci Educ. 2024 Nov 13;35(1):343–50. doi: 10.1007/s40670-024-02191-w (PMC11933490; doi:10.1007/s40670-024-02191-w)
Supplement: Supplementary file 7 — G. Observer Feedback Tool: A rubric to use during the HEEADSSS role play activity. Student not engaged in “doctor” or “patient” role should fill out based on “doctor” student’s interview skills (PDF 167 KB) [file 40670_2024_2191_MOESM7_ESM.pdf]

A Novel Pediatric Clinical Skills Curriculum to Prepare Medical Students for Pediatrics Clerkship

Lindsay Podraza, MD[1]; Lauren S. Starnes, MD, MEd[2]; Joseph R. Starnes, MD, MPH[3], Anuj Patel, MD[4]; Rachel K.P. Apple, MD, MPH[5]
Contributor: Lauren Presley, MSN APRN, CPNP-PC[6]

[1] Pediatric Resident, Monroe Carell Jr. Children's Hospital at Vanderbilt, Nashville, TN, USA. ORCID 0000-0002-4926-0001
[2] Pediatric Hospital Medicine Fellow, Monroe Carell Jr. Children's Hospital at Vanderbilt, Nashville, TN, USA. ORCID 0000-0001-7075-9774
[3] Pediatric Cardiology Fellow, Monroe Carell Jr. Children's Hospital at Vanderbilt, Nashville, TN, USA. ORCID 0000-0001-7954-5385
[4] Assistant Professor of Pediatrics, Monroe Carell Jr. Children's Hospital at Vanderbilt, Nashville, TN, USA
[5] Associate Professor of Internal Medicine and Pediatrics, Vanderbilt University Medical Center, Nashville, TN, USA
[6] Pediatric Nurse Practitioner, Newborn Nursery, Vanderbilt University Medical Center, Nashville, TN, USA

Corresponding author: Lindsay Podraza, lindsaypodraza.md@gmail.com

Observer Form

While one student is interviewing another student role-playing as an adolescent patient, the third student should observe the interaction and provide feedback on the interview using the rubric below.

Topic	Performed? (Yes/No)	Comments
Opener		
Begins with a statement of confidentiality		
Home		
Determines where the patient lives and who lives with/visits the patient		
Asks about feeling safe at home		
Asks about guns at home		
Education		
Determines patient's school, current grade, and academic record		
Asks about feeling safe at school		
Determines if patient has trouble with behavior or attendance at school		
Eating/Exercise		
Asks patient about body comfort		
Asks patient about exercise		
Activities/Employment		
Asks an opener question		
Asks about friends		
Asks about after school activities/work		
Drugs		
Asks an opener question		
Asks about patient substance use		
Completes CRAFFT screener		
Suicidality/Depression		

Provides a warning shot		
Asks an opener question		
Discusses SIG E CAPS		
Screens for active and passive suicidal ideation		
Sexuality		
Provides a warning shot		
Determines patient interests		
Determines past or current sexual activity		
Asks about protection and engages in safe sex counseling		
Safety		
Asks an opener		
Explores car safety		
Explores online safety		
Explores history with law enforcement		
Closer		
Thanks the patient		
Provides reassurance about confidentiality		
Provides next steps		

General Feedback:

Topic	Rating<br>(1 = strongly disagree, 4 = strongly agree)
Avoids medical jargon	
Remains non-judgmental during interaction	
Works to create a safe environment for patient	
Uses open-ended questions to allow patient to engage with interview	

Comments:
