# Supplementary material for: A Novel Pediatric Clinical Skills Curriculum to Prepare Medical Students for Pediatrics Clerkship
Source: Med Sci Educ. 2024 Nov 13;35(1):343–50. doi: 10.1007/s40670-024-02191-w (PMC11933490; doi:10.1007/s40670-024-02191-w)
Supplement: Supplementary file 8 — H. Pre-Intervention Survey: This REDCap survey consists of 5 Likert-style confidence questions was sent to students prior to participation in the curriculum, asking them to rank their confidence in pediatrics-relevant examination skills (PDF 144 KB) [file 40670_2024_2191_MOESM8_ESM.pdf]

A Novel Pediatric Clinical Skills Curriculum to Prepare Medical Students for Pediatrics Clerkship

Lindsay Podraza, MD[1]; Lauren S. Starnes, MD, MEd[2]; Joseph R. Starnes, MD, MPH[3], Anuj Patel, MD[4]; Rachel K.P. Apple, MD, MPH[5]
Contributor: Lauren Presley, MSN APRN, CPNP-PC[6]

[1] Pediatric Resident, Monroe Carell Jr. Children's Hospital at Vanderbilt, Nashville, TN, USA. ORCID 0000-0002-4926-0001
[2] Pediatric Hospital Medicine Fellow, Monroe Carell Jr. Children's Hospital at Vanderbilt, Nashville, TN, USA. ORCID 0000-0001-7075-9774
[3] Pediatric Cardiology Fellow, Monroe Carell Jr. Children's Hospital at Vanderbilt, Nashville, TN, USA. ORCID 0000-0001-7954-5385
[4] Assistant Professor of Pediatrics, Monroe Carell Jr. Children's Hospital at Vanderbilt, Nashville, TN, USA
[5] Associate Professor of Internal Medicine and Pediatrics, Vanderbilt University Medical Center, Nashville, TN, USA
[6] Pediatric Nurse Practitioner, Newborn Nursery, Vanderbilt University Medical Center, Nashville, TN, USA

Corresponding author: Lindsay Podraza, lindsaypodraza.md@gmail.com

Physical Diagnosis: Pediatrics | Pre-Participation Survey

Educational Objectives:
After participation in this curriculum, learners will be able to:
1. Feel confident systematically examining a healthy newborn/infant
2. Feel confident systematically examining a healthy child
3. Feel confident recognizing normal newborn/infant examination findings
4. Feel confident recognizing normal child examination findings
5. Feel confident identifying components included in a HEEADSSS assessment

1.) I feel confident in performing a physical examination of a newborn/infant.

- o Strongly Agree
- o Agree
- o Disagree
- o Strongly Disagree

2.) I feel confident in identifying normal newborn/infant examination findings.

- o Strongly Agree
- o Agree
- o Disagree
- o Strongly Disagree

3.) I feel confident in performing a physical examination of a child.

- o Strongly Agree
- o Agree
- o Disagree
- o Strongly Disagree

4.) I feel confident in my knowledge of the components of a HEEADSSS assessment.

- o Strongly Agree
- o Agree
- o Disagree
- o Strongly Disagree

5.) I feel confident performing a HEEADSSS assessment on an adolescent patient.

- o Strongly Agree
- o Agree
- o Disagree
- o Strongly Disagree
