# Supplementary material for: A Novel Pediatric Clinical Skills Curriculum to Prepare Medical Students for Pediatrics Clerkship
Source: Med Sci Educ. 2024 Nov 13;35(1):343–50. doi: 10.1007/s40670-024-02191-w (PMC11933490; doi:10.1007/s40670-024-02191-w)
Supplement: Supplementary file 9 — I. Post-Intervention Survey: This REDCap survey consists of the same Likert-style confidence questions as the pre-intervention survey, in addition to 5 additional questions on qualitative feedback and participation in the optional after-hours activity. This was sent to students after participation in the curriculum (PDF 157 KB) [file 40670_2024_2191_MOESM9_ESM.pdf]

A Novel Pediatric Clinical Skills Curriculum to Prepare Medical Students for Pediatrics Clerkship

Lindsay Podraza, MD[1]; Lauren S. Starnes, MD, MEd[2]; Joseph R. Starnes, MD, MPH[3], Anuj Patel, MD[4]; Rachel K.P. Apple, MD, MPH[5]
Contributor: Lauren Presley, MSN APRN, CPNP-PC[6]

[1] Pediatric Resident, Monroe Carell Jr. Children's Hospital at Vanderbilt, Nashville, TN, USA. ORCID 0000-0002-4926-0001
[2] Pediatric Hospital Medicine Fellow, Monroe Carell Jr. Children's Hospital at Vanderbilt, Nashville, TN, USA. ORCID 0000-0001-7075-9774
[3] Pediatric Cardiology Fellow, Monroe Carell Jr. Children's Hospital at Vanderbilt, Nashville, TN, USA. ORCID 0000-0001-7954-5385
[4] Assistant Professor of Pediatrics, Monroe Carell Jr. Children's Hospital at Vanderbilt, Nashville, TN, USA
[5] Associate Professor of Internal Medicine and Pediatrics, Vanderbilt University Medical Center, Nashville, TN, USA
[6] Pediatric Nurse Practitioner, Newborn Nursery, Vanderbilt University Medical Center, Nashville, TN, USA

Corresponding author: Lindsay Podraza, lindsaypodraza.md@gmail.com

Physical Diagnosis: Pediatrics | Post-Participation Survey

Educational Objectives:
After participation in this curriculum, learners will be able to:
1. Feel confident systematically examining a healthy newborn/infant
2. Feel confident systematically examining a healthy child
3. Feel confident recognizing normal newborn/infant examination findings
4. Feel confident recognizing normal child examination findings
5. Feel confident identifying components included in a HEEADSSS assessment

1.) I feel confident in performing a physical examination of a newborn/infant.

- Strongly Agree
- Agree
- Disagree
- Strongly Disagree

2.) I feel confident in identifying normal newborn/infant examination findings.

- Strongly Agree
- Agree
- Disagree
- Strongly Disagree

3.) I feel confident in performing a physical examination of a child.

- Strongly Agree
- Agree
- Disagree
- Strongly Disagree

4.) I feel confident in my knowledge of the components of a HEEADSSS assessment.

- Strongly Agree
- Agree
- Disagree
- Strongly Disagree

5.) I feel confident performing a HEEADSSS assessment on an adolescent patient.

- Strongly Agree
- Agree
- Disagree
- Strongly Disagree

6.) I feel that the learning objectives of these sessions were met (listed at top of page).
- Strongly Agree

- Agree
- Disagree
- Strongly Disagree

7.) I was satisfied with the relevance of the pediatrics PDX sessions (infant manikin session, bedside teaching, HEEADSSS role play activity, after-hours session) in helping to prepare me for pediatric clerkship next year.

- Strongly Agree
- Agree
- Disagree
- Strongly Disagree

8.) Did you participate in the optional after-hours session in CELA to practice physical exam skills?

- Yes
- No

9.) Please describe the strengths of the following pediatrics PDX sessions:
-Infant exam practice with manikins
-Bedside teaching sessions
-HEEADSSS didactic/role play activity
-Optional after-hours session in CELA

10.) Please describe suggestions for improvement for the following pediatrics PDX sessions:
-Infant exam practice with manikins
-Bedside teaching sessions
-HEEADSSS didactic/role play activity
-Optional after-hours session in CELA

